# Male *BRCA* mutation carriers: clinical characteristics and cancer spectrum

**DOI:** 10.1186/s12885-018-4098-y

**Published:** 2018-02-13

**Authors:** Mohammed Ibrahim, Siddhartha Yadav, Foluso Ogunleye, Dana Zakalik

**Affiliations:** 1 Department of Hematology/Oncology, Beaumont Health, 3577 W 13 Mile Rd., Ste. 202a, Royal Oak, MI 48073 USA; 2 Oakland University William Beaumont School of Medicine, 2200 N Squirrel Rd, Rochester, MI 48309 USA; 3 Hematology-Oncology Fellowship Program, Mayo Clinic, 200 First Street SW, Rochester, MN 55905 USA; 4 Nancy and James Grosfeld Cancer Genetics Center, Beaumont Health, 3577 W 13 Mile Rd., Ste. 140, Royal Oak, MI 48073 USA

**Keywords:** *BRCA* mutations, Male breast cancer, Prostate cancer, Melanoma, Survival

## Abstract

**Background:**

Mutations in *BRCA*1 and *BRCA*2 (*BRCA*1/2) genes are associated with an increased risk of breast and ovarian cancers in women. The cancer characteristics of men with *BRCA*1/2 mutations are less well studied. This study describes the unique cancer characteristics of male *BRCA*1/2 mutation carriers at our institution.

**Methods:**

We performed a retrospective chart review on male patients who were seen between January 2004 and December 2014 and tested positive for a *BRCA*1/2 mutation. We evaluated clinical characteristics, pathology findings, treatment selection and survival.

**Results:**

A total of 102 male patients were identified who tested positive for a *BRCA*1/2 deleterious mutation. Of these 102 patients, 33 (32%) had a diagnosis of cancer. Of these 33 patients with cancer, the majority (20 patients) were found to carry a *BRCA*2 mutation. Median age of cancer diagnosis was 65 years (Range: 35-75 years). Of the 33 patients diagnosed with cancer, 8 had two or more cancers, including 1 patient who had 4 cancers. Prostate cancer was the most commonly diagnosed cancer, seen in 13 patients, 11 of whom were *BRCA*2 positive. These cancers tended to have higher Gleason scores and elevated PSA levels. The majority of these prostate cancer patients were alive and disease free at a median follow-up of 7.4 years. Male breast cancer was the second most common cancer seen in 9 patients, all of whom were *BRCA*2 positive. The majority of these cancers were high grade, hormone receptor positive and associated with lymph node metastases. There were no breast cancer related deaths. Other cancers included bladder cancer, pancreatic cancer, melanoma and other skin cancers.

**Conclusions:**

This study describes the cancer characteristics and outcomes of male *BRCA*1/2 mutation carriers. A third of male *BRCA*1/2 mutation carriers had a diagnosis of cancer. A significant number of patients (mostly *BRCA*2 mutation positive) developed multiple cancers, which may have important implications for cancer screening and prevention. Despite having high grade histology and advanced stage at diagnosis, male *BRCA*1/2 mutation carriers with breast and prostate cancer demonstrated a favorable 5-year survival.

## Background

*BRCA*1 and *BRCA*2 *(BRCA*1/2 hereafter) are tumor suppressor genes involved in DNA repair and maintenance of genomic stability. Mutations in *BRCA*1/2 genes account for 20 to 25% of all hereditary breast cancers [1] and about 5–10% of all breast cancers [2]. Pathogenic *BRCA*1/2 mutations are highly penetrant mutations that are inherited in an autosomal dominant fashion and result in a significantly increased risk of breast, ovarian, prostate, melanoma, pancreatic and other cancers [3–7]. While there has been extensive research on the risk of cancer in women with *BRCA*1/2 mutations, the cancer characteristics of men with *BRCA*1/2 mutations have not been well studied.

Men with *BRCA*1/2 mutations are at increased risk for breast, prostate, pancreatic and other cancers [3–5, 8]. Male breast cancer is rare in the general population with a lifetime risk of 0.1%, although the risk is significantly increased to 7-8% with *BRCA*2 mutation and 1% with a *BRCA*1 mutation [3, 4]. Up to 14% of men diagnosed with breast cancer are found to harbor a *BRCA*2 mutation [8]. Two prior studies have reported worse prognosis in male breast cancer patients with a deleterious *BRCA*1/2 mutation compared to those without a mutation [9, 10]. However, both studies were limited by their small sample size. There is limited knowledge about the clinical characteristics of male *BRCA*1/2 mutation carriers.

The risk of prostate cancer is up to fivefold higher in *BRCA*2 mutation carriers [5, 6]. *BRCA*1/2 mutation associated prostate cancers have been reported to be more aggressive and associated with a worse survival compared to *BRCA* wild type cancers [11–14]. Castro et al. reported on 79 patients with prostate cancer who were positive for a *BRCA*1/2 mutation and found that these patients frequently presented with higher Gleason scores (≥ 8), higher T stage (T3 or T4), nodal involvement and metastases at diagnosis [12].

*BRCA*1/2 associated breast cancers have been known to respond better to platinum based chemotherapy [15]. Knowledge of *BRCA*1/2 mutation status can have therapeutic implications which can impact survival. PARP (poly adenosine diphosphate-ribose polymerase) inhibitors are being used increasingly in advanced breast and ovarian cancer in women [16–19]. These findings are expanding to treatment of male *BRCA* associated cancers. A recent study demonstrated a significant response to a PARP inhibitor, Olaparib, in patients with advanced castrate resistant prostate cancer with *BRCA* mutations [20]. Whether these therapeutic agents and the knowledge of *BRCA* mutation status translate to better outcomes in men is not yet known.

In summary, while little is known about the clinical characteristics and outcomes of male *BRCA* mutation carriers, there is a growing imperative to expand our understanding of this unique population. We present an analysis of male *BRCA* mutation carriers from our cancer genetics clinic database and describe their unique features and outcomes.

## Methods

After Institutional Review Board approval, we performed a retrospective chart review on male *BRCA*1/2 mutation carriers identified at the Nancy and James Grosfeld Cancer Genetics Center from January 2004 to December 2014. Patients with a pathogenic or likely pathogenic *BRCA*1/2 mutation were included in the study, while those with variants of undetermined significance were excluded. We used CLIA certified commercial laboratories such as Ambry, Myriad and Invitae, (which are widely used in academic centers), to obtain the information about pathogenicity, which was also individually verified for concordance using databases such as ClinVar. We looked at the demographic information including their age, race, ethnicity and reason for testing. We then studied the subset of male *BRCA*1/2 mutation carriers who were diagnosed with cancer, either prior to or after the testing was performed. We evaluated the type of cancer, clinical staging, histo-pathologic information, treatment and survival. We used SPSS version- 21 statistical software for data analysis.

## Results

### Demographics

A total of 102 male patients were identified who tested positive for a *BRCA*1/2 deleterious mutation. Fifty-three (52%) of these 102 patients were positive for a *BRCA*1 mutation and 49 (48%) were positive for a *BRCA*2 mutation. The median age at the time of testing was 55 years (Range: 19-85 years).

The race and ethnicities of these 102 individuals are included in Table 1. The majority were Caucasian, of whom a quarter were Ashkenazi Jewish.

**Table 1:** Ethnicities of male *BRCA* mutation carriers

Race/ethnicity	Total *BRCA* mutation carriers (%)	Diagnosed with cancer (%)
Caucasian	97 (95)	32 (97)
Ashkenazi Jewish	25 (25)	10 (30)
Iraqi	3 (3)	3 (9)
Greek	1 (1)	1 (3)
Lebanese	1 (1)	1 (3)
Hispanic	1 (1)	1 (3)
African American	2 (2)	1 (3)
Asian	3 (3)	–
Indian	1 (1)	–
Filipino	2 (2)	–
Total	102	33

### Genetic testing

The most common reasons for testing were a family history of breast or ovarian cancer or a known *BRCA*1/2 mutation in the family, which was present in 98 of the 102 patients. A new diagnosis of male breast cancer prompted *BRCA*1/2 testing in the 4 individuals without a family history of breast and ovarian cancer.

Of the non-Ashkenazi Jewish population, 66 patients (86%) underwent single site testing, 9 patients (12%) had full sequence testing and 2 (2%) had multi-gene panel testing. Of the 25 Ashkenazi Jewish patients who tested positive for a *BRCA*1/2 mutation, the majority were identified using the 3 site Ashkenazi Jewish panel, while an additional 3 patients were identified to carry an elsewhere mutation, after being tested negative for the 3 site Ashkenazi Jewish panel.

### Cancer characteristics

**Fig. 1:**
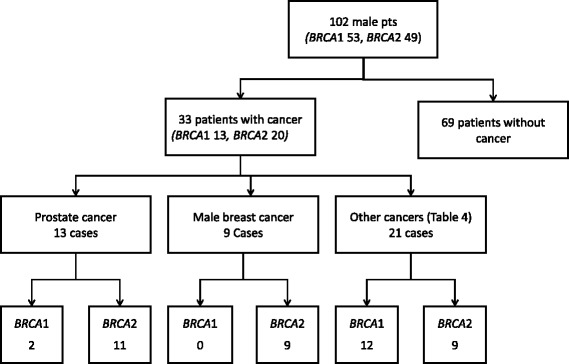
*BRCA* mutation carriers and diagnosis of cancer

Of the 102 patients who tested positive for a *BRCA*1/2 mutation, 33 (32%) had a diagnosis of cancer (Fig. 1). Of these 33 patients with cancer, the majority (20 patients) were found to carry a *BRCA2* mutation. Median age of diagnosis with cancer was 65 years (Range: 35-75 years). A total of 44 cancers were diagnosed in these 33 patients.

**Fig. 2:**
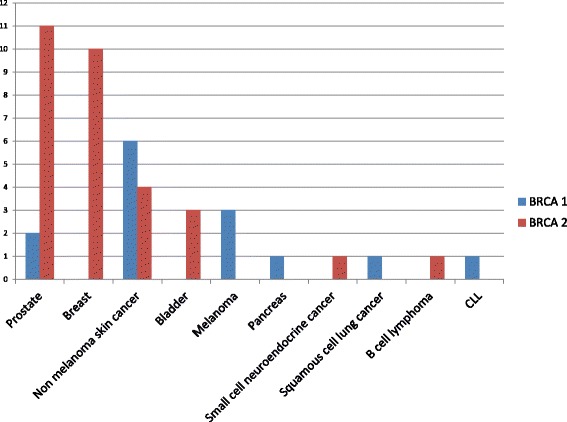
Types of cancers diagnosed in 33 male *BRCA* mutation carriers

Of the 33 patients diagnosed with cancer, the majority (25 patients) had 1 cancer. A quarter of patients (8 patients) had more than 1 cancer. Of these 8 patients, 6 had 2 cancers, 1 patient had 3 cancers (breast, prostate and lymphoma) and 1 patient had 4 different cancers (breast cancer, DCIS, prostate and small cell neuroendocrine cancer) (Figs. 1 and 2). Of the 8 patients diagnosed with multiple cancers, the majority (7 patients) had a *BRCA*2 mutation. A third (7 patients) of the 20 *BRCA*2 mutated patients with cancer had multiple cancers.

Prostate cancer was the most commonly diagnosed cancer, followed by male breast cancer, bladder cancer, melanoma and other skin cancers (Fig. 1, Tables 2 and 3). Ten patients had a diagnosis of non-melanoma skin cancer. Other cancers diagnosed in this group included pancreatic cancer, small cell neuroendocrine cancer, squamous cell lung cancer and lymphomas (Table 4 and Fig. 2).

**Table 2:** Cancer characteristics of male *BRCA* mutation carriers with prostate cancer

Patient ID	BRCA Gene	Mutation	Age at diagnosis	PSA	Stage	TNM	Gleason	Surgery	RT	ADT	Status	F/U (Yrs)
15	1	c.181 T > G	51	N.A	II	T2Nx	7	yes	N.A	N.A	Alive, NED	5.4
10	1	c.4096 + 1 G > A	80	N.A	NA	N.A	N.A	NA	yes	N.A	Alive, NED	13
4	2	c.100 G > T	72	N.A	NA	N.A	6	Yes, RP	NA	NA	Alive, NED	10.1
3	2	c.1813dupA	58	7.9	I	T1 N0	6	NA	yes	N.A	Alive, NED	15.6
5	2	c.2808del4	66	N.A	N.A	N.A	N.A	N.A	yes	N.A	Alive, NED	5
1	2	c.2808del4	67	N.A	early	N.A	6	no	yes, EBRT	Leuprolide-3 years	Alive, NED	7.1
12	2	c.5576del4	75	20.84	IV	N.A	9	no	Palliative RT to spine	Leuprolide	Expired	0.3
16	2	c.5576del4	70	N.A	III	T3 N0	8	yes, RP	N.A	Leuprolide	Alive, NED	10
14	2	c.5946delT	64	2.7	N.A	N.A	N.A	yes, RP	N.A	N.A	N.A	N.A
7	2	c.5946delT	74	N.A	N.A	N.A	7	yes, RP	N.A	Leuprolide	Alive, NED	8.1
13	2	c.7558 C > T	67	N.A	II	T2,N0	7	no	brachytherapy	N.A	Alive, NED	7.6
11	2	c.9317G > A	65	N.A	II	T2 N0	7	Yes,RP	N.A	N.A	Alive, NED	5.2
6	2	c.6333_6337delGAGAA	68	1497	IV	N.A	N.A	N.A	N.A	Bicalutamide, Leuprolide, Abiraterone	Expired	2.2

*RT* radiation therapy, *RP* Radical prostatectomy, *ADT* androgen deprivation therapy, *NED* no evidence of disease, *NA* not available, *PSA* value in ng/ml, cutoff value 4 ng/ml

**Table 3:** Cancer characteristics of male *BRCA* mutation carriers with breast cancer

ID	BRCA Gene	Mutation	Age at diagnosis	Size (cm)	LN	Stage	TNM stage	Grade	ER %	PR %	HER-2	Surgery	Chemo	RT	Status	F/U (yrs)
4	*2*	c.100G > T	75	1.4	neg	I	T1N0M0	2	92	18	pos	M	Yes, Trastuzumab	no	Alive, NED	5.7
8	*2*	c.736del20	52	N.A	N.A	N.A	N.A	3	97	87	Border-line	M	yes, TCH	yes	Alive	5.6
3	*2*	c.1813dupA	58	1.3	pos	II	T2N1M0	3	59	50	neg	M	yes, AC	yes	Expired	15.8
5	*2*	c.2808del4	67	1.7	pos	III	T1N3M0	3	100	89	pos	M	yes, ACTH	yes	Alive, NED	3.4
1	*2*	c.2808del4	68	1.6	pos	II	T2N1M0	3	90	40	neg	M	Yes, AC-T	yes	Alive, NED	7.0
7	*2*	c.5946del T	62	N.A	pos	II	N.A	3	90	N.A	N.A	M	Yes,CMF	yes	Alive, NED	21.0
9	*2*	c.5946delT	67	N.A	N.A	N.A	N.A	N.A	N.A	N.A	N.A	N.A	N.A	N.A	N.A	N.A
2	*2*	c.6676_6677delGA	62	2.5	pos	II	T2N1M0	2	100	37	pos	M	yes, TCHP	no	Alive, NED	1.8
6	*2*	c.6333_6337delGAGAA	68	6.0	pos	III	T4N2M0	3	97	29	neg	M	N.A	N.A	Expired	2.3

*LN* lymph node status, *RT* radiation therapy, *M* mastectomy, *AC-T* Doxorubicin, Cyclophosphamide and Paclitaxel, *TCHP* Docetaxel, Carboplatin, Trastuzumab and Pertuzumab, *AC* Doxorubicin and Cyclophosphamide, *ACTH* Doxorubicin, Cyclophosphamide, Paclitaxel and Trastuzumab, *CMF* Cyclophosphamide, Methotrexate and Fluorouracil, *TCH* Docetaxel, Carboplatin and Trastuzumab, *NED* no evidence of disease, *F/U* follow-up, *NA* not available, *neg* negative, *pos* positive

**Table 4:** Other cancers diagnosed in male *BRCA* mutation carriers

Cancer	Patient ID	BRCA gene	*Mutation*	Age at dx	Stage	Surgery	Chemotherapy	RT	Status	F/U (Yrs)
Melanoma *N* = 3	19	1	c.66dupA	36	Early	yes	no	no	N.A	N.A
	17	1	c.68delAG	62	IV	N.A	Yes (Cisplatin, PARP inhibitor clinical trial)	yes	expired	0.9
	18	1	del exons 1-7	67	early	yes	no	no	N.A	N.A
Bladder N = 3	20	2	c.5576del4	47	early	Yes (TURBT)	no	no	N.A	N.A
	22	2	c.5782 G > T	70	IV	Yes (nephro-ureterectomy)	Yes (Carboplatin, Paclitaxel, Pemetrexed	yes	expired	2.4
	21	2	c.5946delT	59	early	Yes (TURBT)	no	no	Alive, NED	4.7
Squamous cell lung cancer	23	1	c.5123C > A	87	III	No (declined)	No (declined)	No (declined)	expired	1.1
Neuroendocrine carcinoma	3	2	c.1813dupA	73	IV	no	Yes (Carboplatin, Etoposide)	no	expired	1.4
Pancreas	24	1	c.2475delC	60	IV	N.A	Yes (Gemcitabine, Carboplatin, FOLFOX)	no	Expired	4.2
Lymphoma	1	2	c.2808del4	68	N.A	N.A	N.A	N.A	N.A	N.A
CLL	25	1	c.68delAG	50	N.A	N.A	N.A	N.A	alive	1.1
Other Cancers *N* = 10	28	1	c.68delAG	65	early	yes	N.A	N.A	alive	N.A
	26	1	c.2475delC	55	early	yes	N.A	N.A	N.A	N.A
	30	1	c.4524G > A	55	early	yes	N.A	N.A	N.A	N.A
	23	1	c.5123C > A	74	early	yes	N.A	N.A	N.A	N.A
	29	1	c.5266dupC	37	early	yes	N.A	N,A	N.A	N.A
	31	1	del exons 13-15	NA	early	yes	N.A	N.A	N.A	N.A
	22	2	c.5782G > T	58	early	yes	N.A	N.A	N.A	N.A
	33	2	c.9196C > T	60	early	yes	N.A	N.A	N.A	N.A
	27	2	c.9382C > T	35	early	yes	N.A	N.A	N.A	N.A
	32	2	1881 delC	70	early	yes	N.A	N.A	N.A	N.A

*RT* radiation therapy, *F/U* follow-up, *TURBT* transurethral resection of bladder tumor, *FOLFOX* Infusional fluorouracil, leukovorin and oxaliplatin, *N.A* not available

#### Prostate cancer

Thirteen patients had a diagnosis of prostate cancer (Table 2). The majority (11) of these patients had a *BRCA*2 mutation. Median age at diagnosis with prostate cancer was 68 years (Range: 51 to 80 years). Of the 4 patients for whom PSA (prostate specific antigen) data was available, the median PSA level at diagnosis was 14.37 ng/ml (Range: 2.7 to 1497 ng/ml). Of the 8 patients for whom staging information was available, 3 had stage II disease, 2 patients had stage I disease, 2 had stage IV disease and 1 patient had stage III disease. Of the 9 patients for whom Gleason score was available, the median Gleason score was 7 (Range: 6-9). Most patients were treated with either radical prostatectomy (6 patients) or primary radiation therapy (5 patients). The remaining 2 patients had stage IV disease and were treated with palliative chemotherapy (Docetaxel), androgen deprivation therapy (ADT) and palliative radiation therapy to the spine.

**Fig. 3:**
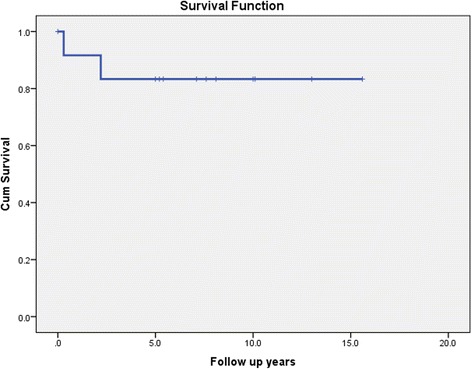
Kaplan-Meier curve showing overall survival in male *BRCA* mutation carriers with prostate cancer

Survival data was available for 12 patients. Two patients died during a median follow-up of 7.4 years (Range: 0.3 - 15.6 years). One patient died 4 months after diagnosis from stage IV disease with extensive bone, liver and lung metastases and another patient died at 2.2 years, also from extensive bone and liver metastases. The 1- and 5-year survival was 91.6% and 83.3% respectively (Fig. 3). The 5- year prostate cancer specific survival was 83.3%.

#### Male breast cancer

**Fig. 4:**
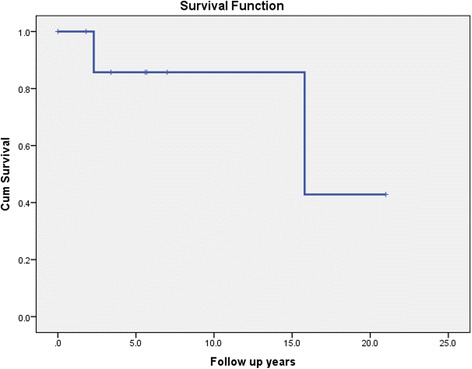
Kaplan-Meier curve showing overall survival in male *BRCA* mutation carriers with breast cancer

Nine patients had a diagnosis of male breast cancer (Table 3). All of these 9 patients had a *BRCA*2 mutation. Median age at breast cancer diagnosis was 67 years (Range: 52-75 years). All of the 8 patients for whom pathology was available, had infiltrating ductal carcinomas and one of these also had a contralateral DCIS (ductal carcinoma in situ). All of these 8 patients had hormone receptor ER (estrogen receptor) positive cancers and 6 had high grade histology. Of the 7 patients for whom lymph node status was available, 6 patients had lymph node metastases at diagnosis. Staging information was available for 7 patients. Four of these 7 patients had stage II disease, 1 patient had stage I disease and 2 patients had stage III disease. Four out of the 7 patients for whom human epidermal growth factor receptor (HER-2) status was available, were HER − 2 positive. Eight patients had available surgical treatment data and all eight patients underwent a mastectomy. Five of the 9 breast cancer patients received adjuvant radiation therapy. Seven patients received chemotherapy, as depicted in Table 3. All but 1 patient took Tamoxifen. Two patients died during a median follow-up of 5.6 years. One patient died 15.8 years after diagnosis, from small cell neuroendocrine cancer and another patient died 2.3 years after diagnosis from widely metastatic prostate cancer. There were no breast cancer associated deaths. The 1- and 5- year survival was 100% and 83.3% respectively (Fig. 4). The 5- year breast cancer specific survival was 100%.

#### Other cancers

Three patients were diagnosed with malignant melanoma. Ten patients were diagnosed with non-melanoma skin cancers (Table 4). One of the 3 patients with melanoma had an aggressive anal melanoma, which presented with stage IV disease. This patient was treated with multiple lines of chemotherapy including Cisplatin as well as a PARP inhibitor. He initially had a good response to the PARP inhibitor, but eventually succumbed to the disease. Three patients were diagnosed with bladder carcinoma. One patient was diagnosed with metastatic pancreatico-biliary adenocarcinoma. One patient had small cell neuroendocrine carcinoma of liver and was treated with Carboplatin and Etoposide chemotherapy. One patient was diagnosed with squamous cell lung carcinoma and elected for hospice care in view of advanced age and multiple co-morbidities.

## Discussion

This study represents one of the largest retrospective studies of cancers in male *BRCA*1/2 mutation carriers, consisting of 102 male patients who were identified in a clinical cohort. Given the paucity of recent literature on cancer characteristics in male *BRCA* mutation carriers, this study will help enhance our understanding of the cancer characteristics in this unique population. The most common reason for testing among our male population was a family history of breast and ovarian cancer, which was present in 98 (96%) of the 102 patients. Only 4 of the 102 patients were tested after their own cancer diagnosis. It is important to be aware that male breast cancer may be the first manifestation of a *BRCA* mutation in a family, reinforcing the NCCN recommendation that every male breast cancer patient should be tested for a *BRCA* mutation [21].

Despite having a family history of breast and ovarian cancer, the majority of males in our study underwent single site testing, suggesting that testing typically occurred after another family member was identified with a mutation. Other studies have also shown that males are less likely to test for *BRCA* mutations [22–24]. Despite autosomal inheritance, males tend to be less likely to be the first in the family to test. One possible reason for this observation is physician bias regarding *BRCA* impacting women given the well-known association of *BRCA* mutation with female cancers. Other reasons include lack of awareness that breast cancer can affect men or that breast cancer genes can be transmitted by males and also less willingness of males with regard to genetic testing [22–24].

Almost a third of male patients with a *BRCA* mutation had a diagnosis of cancer, but only 4 patients were tested because of the cancer diagnosis. The median age of diagnosis of any cancer was 65 years. There was no identifiable pattern of mutations with respect to the region of the gene or the type of mutation, among the people unaffected by cancer, compared to those who developed a cancer. Of the 20 *BRCA*2 mutation carriers with cancer, over a third (7 patients) had multiple cancers. Physicians need to be vigilant to the possibility of synchronous or metachronous development of new cancers in the *BRCA* mutation carriers, especially those with a *BRCA*2 mutation. Prostate cancer was the most common cancer diagnosed in this population followed by male breast cancer, skin cancer, bladder cancer and others.

The majority of cancers were seen in *BRCA*2 mutation carriers, which is consistent with previously reported data [3–7]. The majority of *BRCA* associated male breast cancers were high grade, hormone receptor positive and associated with lymph node metastases, which is also consistent with previously reported data [25] It is interesting to note that all of the 7 patients for whom chemotherapy data was available, received chemotherapy. There were no breast cancer related deaths. The impact of chemotherapy on the excellent survival noted in this cohort of patients is intriguing. Homologous recombination repair deficiency seen in *BRCA* mutated cancers confers higher sensitivity to chemotherapy, especially to DNA damaging agents such as platinum based therapies, due to impaired ability to repair double strand breaks. More than two thirds of patients with breast cancer were alive with no evidence of disease, at a median follow-up of 5.6 years.

Although limited by small size, our study shows that *BRCA* mutation carriers with prostate cancer had higher Gleason scores, elevated PSA levels and more advanced stage disease, consistent with prior reports [12–14]. The median age of diagnosis with prostate cancer was 68 years, lending support to the recent change in NCCN guidelines to increase the age of prostate cancer screening in *BRCA* mutation carriers from 40 to 45 years [21].

Of the less common cancers, we observed 1 pancreatic cancer that was interestingly identified in a *BRCA*1 mutation carrier. The risk of uveal melanoma (a rare type of melanoma) has been reported to be higher in *BRCA*2 mutation carriers [7]. We had 3 patients who were diagnosed with melanoma in our study, but neither one of them had an uveal melanoma. One patient had an aggressive stage IV anal melanoma and demonstrated an excellent initial response to a PARP inhibitor on a clinical trial. This observation supports further research into PARP inhibitors in other *BRCA* related cancers.

Our study had a few limitations, including being a single institution retrospective analysis as well as limited access to treatment records, and somewhat short follow-up.

## Conclusions

This study represents one of the largest studies describing the cancer characteristics of male *BRCA* mutation carriers. We demonstrated that the majority of cancers seen were breast and prostate with high grade histology and more advanced stages. Favorable impact of systemic chemotherapy may explain the excellent survival in the male breast cancer group. Additional studies are needed with large numbers of patients to better understand the cancer characteristics of male *BRCA* mutation carriers.

## Abbreviations

AC: Doxorubicin and cyclophosphamide
AC-T: Doxorubicin, cyclophosphamide and paclitaxel
ACTH: Doxorubicin, cyclophosphamide, paclitaxel and trastuzumab
ADT: Androgen deprivation therapy
CMF: Cyclophosphamide, methotrexate and fluorouracil
DCIS: Ductal carcinoma in situ
ER: Estrogen receptor
F/U: Follow-up
FOLFOX: Infusional fluorouracil, leucovorin and oxaliplatin
HER-2: Human epidermal growth factor receptor-2
LN: Lymph node
M: Mastectomy
NED: No evidence of disease
PARP: Poly adenosine diphosphate-ribose polymerase
PSA: Prostate specific antigen
RT: Radiation therapy
TCH: Docetaxel, carboplatin and trastuzumab
TCHP: Docetaxel, carboplatin, trastuzumab and pertuzumab
TURBT: Transurethral resection of bladder tumor
